# Advancing Sustainable Agriculture: Molecular and Physiological Insights into Rapeseed Responsiveness to Organic Amendment Fertilization

**DOI:** 10.3390/plants14182937

**Published:** 2025-09-22

**Authors:** Pedro J. Picazo, María Ancín, Bertrand Gakière, Françoise Gilard, David Soba, Angie L. Gámez, Diane Houdusse, Iker Aranjuelo

**Affiliations:** 1 Instituto de Agrobiotecnología (IdAB), Consejo Superior de Investigaciones Científicas (CSIC)-Gobierno de Navarra, Avenida Pamplona 123, 31192 Mutilva, Spain; pedro.picazo@csic.es (P.J.P.); angie.gamez@csic.es (A.L.G.); 2 Plateforme Metabolisme-Métabolome, Université Paris-Saclay, 91190 Gif sur Yvette, Francefrancoise.gilard@universite-paris-saclay.fr (F.G.); 3 CNRS, INRAE, Université Paris-Cité, 91190 Gif sur Yvette, France; 4 Institute of Plant Sciences Paris-Saclay (IPS2), Université Evry, 91190 Gif sur Yvette, France; 5 Cooperl Environnement, 7 Rue des Blossières Maroué, 22400 Lamballe-Armor, France

**Keywords:** amendment, growth, metabolism, oxidative stress, photosynthesis, rapeseed

## Abstract

The widespread use of chemical fertilizers has raised concerns because of their environmental impacts, including soil degradation, water contamination, and biodiversity loss. The integration of organic amendments into agricultural systems provides a sustainable alternative. This study investigates the molecular and physiological traits underlying rapeseed responses to organic amendments based on poultry and plant material mixed with the soil. Plant growth, CO_2_ assimilation, metabolic, proteomic, and soil microbial analyses were performed. Results show a significant stimulation of plant growth (100%) and leaf biomass (200%) following amendment application. This response is attributed to enhanced efficiency in light energy use for CO_2_ fixation, increased carbohydrate and amino acid production, and improved biomass and yield. Increased upregulation of proteins and antioxidant metabolites such as abscisic acid (ABA) indicates an enhanced capacity to cope with oxidative stress. The amendments activated metabolic mechanisms that improved redox balance and homeostasis, including more efficient light energy use and enhanced antioxidant synthesis. Furthermore, the organic amendments promoted Actinobacteria in the soil, contributing to improved soil quality. These metabolic responses may enhance plant resilience against oxidative stress and environmental fluctuations. These findings highlight promising strategies to enhance crop productivity and resilience, advancing sustainable agriculture and strengthening future food security.

## 1. Introduction

The Green Revolution, a transformative movement in agriculture of the mid-20th century, was founded on several core principles aimed at significantly boosting global food production. Some of their main approaches were the adoption of high-yielding crop varieties, modern irrigation systems, and the extensive use of chemical fertilizers and pesticides [1]. These initiatives aimed to enhance agricultural productivity, improve crop yields, and address food scarcity associated with a rapidly growing global population [2]. At present, a substantial share of agricultural production depends on using chemical fertilizers to meet society’s dietary needs [3]. However, this reliance on intensive farming practices has raised concerns about environmental sustainability, including soil degradation, water pollution, and biodiversity loss [4,5].

Current crop production systems face the challenge of improving both productivity and quality to meet the increasing food demands of a growing global population. Furthermore, the consequences of climate change for agricultural ecosystems are expected to intensify, as rising temperatures and reduced water availability are projected to negatively impact crop production systems worldwide [6]. Within this context, organic manure, including plant biomass and animal waste, has been described as serving a vital role as a soil amendment. Organic amendments have been reported to play an important role in nutrient recycling and the maintenance of plant health [7]. Organic amendments, derived from plant and animal sources, act as natural fertilizers and can originate from diverse materials, including agricultural residues such as plant biomass and livestock manure, as well as industrial by-products and municipal sludge. Among the most common examples are animal and green manures, which not only improve the soil structure but also enhance microbial activity and increase nutrient availability [8,9].

Previous studies have shown that the application of organic amendments positively affects plant growth. Research conducted in different regions worldwide indicates that the use of animal manures increases the productivity of diverse crops [10,11]. For instance, one study [12] that compared the effects of different organic sources (animal manures vs. plant residues) on rice (*Oryza sativa* L.) productivity reported that manure application increased growth parameters, yield, and yield components. On average, rice grown with animal manures produced approximately a 20% higher grain yield than rice grown with crop residues. In this context, studies conducted with plants subjected to stress conditions [13,14,15] revealed that the amendment significantly improve plant growth by enhancing stress tolerance. According to those studies, organic fertilizer application ameliorates declines in chlorophyll content, increased photosynthesis, and reduced reactive oxygen species (ROS) accumulation and lipid peroxidation. Furthermore, as reported by [13], the improved crop performance associated with organic amendments was also linked to the stimulation of antioxidant enzyme activities and non-enzymatic antioxidants under drought conditions, thereby contributing to enhanced plant resilience.

Application of manure on crops enhances the yield through various mechanisms that are linked to soil features. Amendments enhance soil quality by introducing vital carbon compounds necessary for plant growth, increasing organic material levels, and promoting the proliferation of soil microorganisms [8,16]. Moreover, organic amendments are known to have a noteworthy impact in enhancing disease resistance in plants, a process that has been linked to changes in the signaling pathways and the activation of systemic resistance mechanisms [7,17]. Besides enhancing nutrient utilization efficiency and subsequently boosting crop productivity [18], organic amendments are effective in preserving the organic matter levels in agricultural soils while also safeguarding and enhancing soil fertility [19]. This is accomplished through the stimulation of the soil microbial community, thereby supporting soil and plant health.

The extensive reliance on chemical fertilizers in modern agriculture has raised significant environmental concerns, including soil degradation, water pollution, and biodiversity loss. To address these challenges, the incorporation of organic amendments such as compost, biochar, and animal manure into cropping systems has emerged as a promising strategy for promoting sustainable and ecologically sound fertilization practices.

Rapeseed (*Brassica napus* L.) is a major oilseed crop with wide-ranging uses in food, feed, and industry [20]. Despite its agronomic and nutritional importance, its productivity is increasingly affected by climate-related stresses and a strong dependence on soil fertility. Notably, rapeseed has high nutrient demands and shows considerable sensitivity to soil conditions, making it particularly responsive to fertilization strategies [21].

These agronomic characteristics make rapeseed an ideal candidate for evaluating alternative fertilization strategies. Investigating the effects of organic amendments on rapeseed growth and yield not only addresses the environmental concerns associated with chemical fertilizers but also contributes to the development of resilient and eco-friendly agricultural systems.

The current study focuses on the characterization of molecular and physiological traits involved in the responsiveness of rapeseed plants fertilized with organic amendments. These findings have crucial implications for enhancing plant productivity and resilience in agricultural settings and plant physiology.

## 2. Results

### 2.1. Plant Growth and Nitrogen Content

Plant biomass significantly increased with amendment application (100%) compared to control plants; the dry weights of shoots and leaves of treated plants were also significantly higher on average (approximately 200%). Grain yield and root weight tended to increase in treated plants, but the differences were not significant. The treatment also had a significant positive effect on leaf nitrogen content, showing an increase of 80% in treated plants (Table 1).

### 2.2. Gas Exchange and Fluorescence Determination

**Figure 1:**
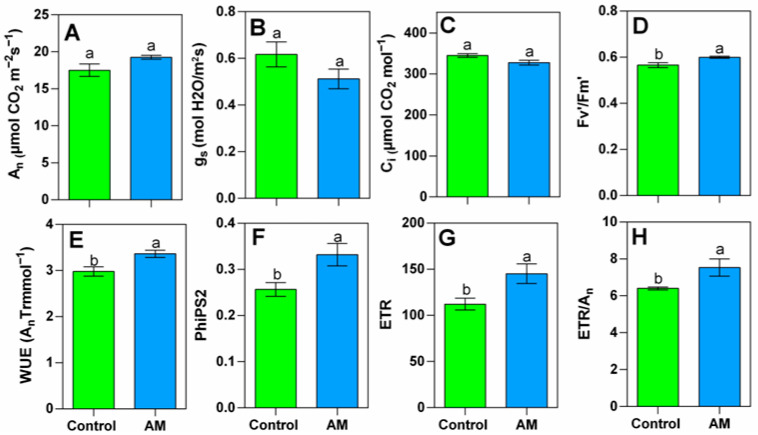
Net [CO_2_] assimilation (**A**), total leaf conductance (**B**), intercellular CO_2_ concentration (**C**), efficiency quantum yield of PSII (**D**), water use efficiency (**E**), PSII efficiency (**F**), electron transport rate (**G**), and electron transport rate divided by net CO_2_ assimilation (**H**) of rapeseed plant growth, with amendment application showed in blue bars (AM) and non-treated plants shown in green bars (Control). Bars are means (*n* = 4), and capped lines are standard errors. Within each graph, bars with different letters indicate that values are significantly different (*p* < 0.05) in *t*-test.

No significant differences in net CO_2_ assimilation (An), total leaf conductance (gs), or intercellular CO_2_ concentration were found between treatments (Figure 1A–C). However, fluorescence parameters were higher in treated plants, with minor changes in values of PSII (Fv’/Fm’), and significant notable changes in photosystem II efficiency (PhiPS2), electron transport rate (ETR), water use efficiency (An/Trmmol), and the ratio of electron transport to assimilation (ETR/AN) in treated plants (Figure 1D–H).

### 2.3. Chlorophyll and Anthocyanin Content

**Figure 2:**
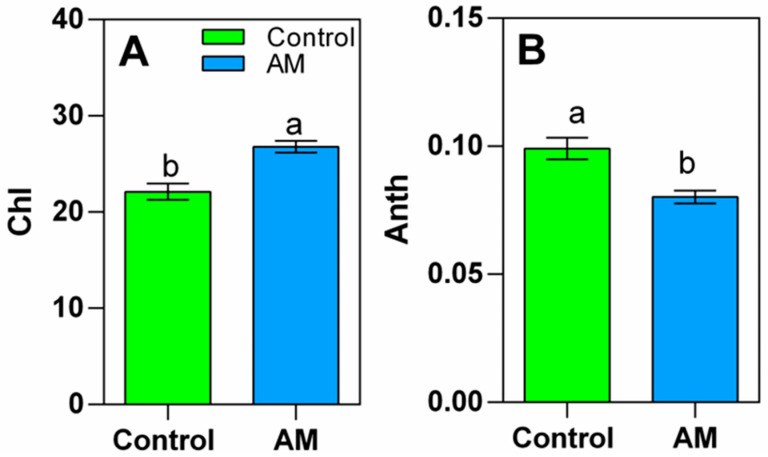
Chlorophyll lead content (**A**) and anthocyanin leaf content (**B**) in Dualex units of rapeseed plant growth, with amendment application shown in blue bars (AM) and non-treated plants shown in green bars (Control). Bars are means (*n* = 4), and capped lines are standard errors. Within each graph, bars with different letters indicate that values are significantly different (*p* < 0.05) in *t*-test.

The chlorophyll leaf content significantly increased by 20% in amended plants (Figure 2A), while the anthocyanin level decreased by the same amount in amended plants compared to control plants (Figure 2B).

### 2.4. Plant Metabolism Analyses

**Figure 3:**
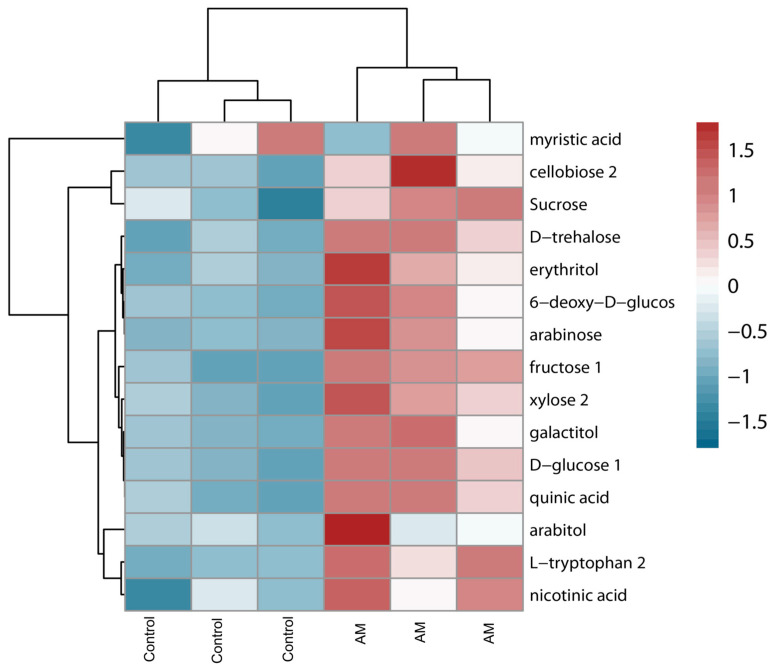
Heatmap of metabolites identified in rapeseed plants under control conditions (Control) and amended application (AM), made in R. Heatmap was arranged using Z-score values of analyzed metabolites, where colors ranging from red (increase) to blue (decrease) are shown in the legend. Hierarchical clustering of both metabolites and samples (*n* = 3) was performed using Euclidean distance and average linkage.

Metabolomic analysis revealed 13 metabolites that accumulated significantly under amendment application. Among these metabolites, carbohydrates such as sucrose, fructose, D-glucose, and 6-deoxy-D-glucose, related to the plant’s energetic status, accumulated in the leaves of amended plants and showed similar clustering patterns. On the other hand, organic acids (quinic acid and nicotinic acid) related to the redox homeostasis and stress signaling also accumulated under amendment application. Additionally, the amendment positively affected structural polysaccharides such as cellobiose, amylose, and arabinose, which are cell wall components. Signal molecules related to plant–microorganism interactions, including osmoprotectants such as trehalose, erythritol, and galactitol, also increased in amended plants (Figure 3). These different metabolites’ accumulation, mainly those that are related to plant structural components, aligns well with previous results of growth enhancement such as biomass and bigger leaves (Table 1).

### 2.5. Proteomic Analyses

**Figure 4:**
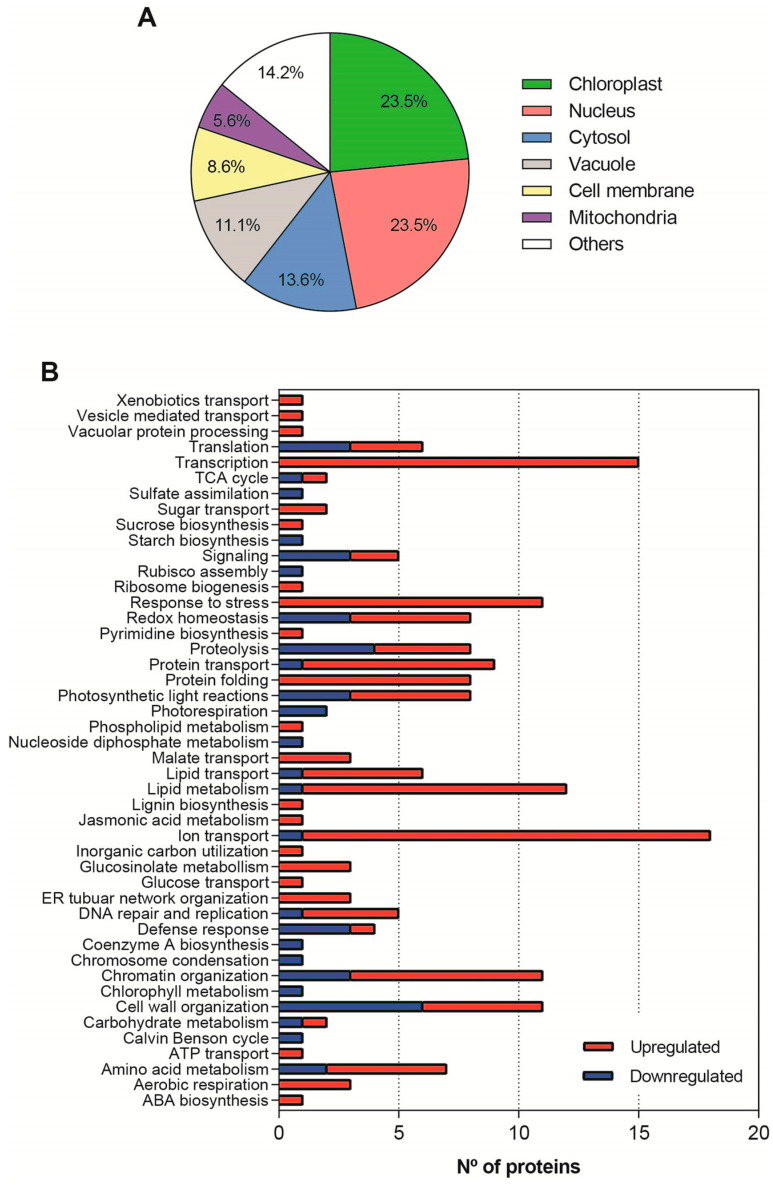
Diagram representing the % of accumulated proteins in different cell organelles (**A**) and histogram of upregulated and downregulated proteins in different metabolic routes (**B**), identified in oilseed rape plants in response to organic amendment fertilization.

**Figure A1:**
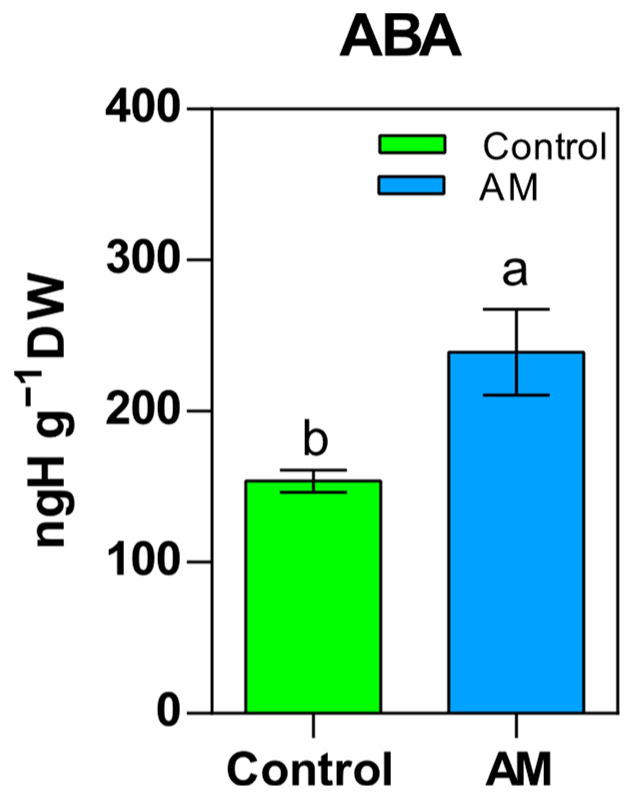
Leaf concentration (ng g^−1^ DW) of abscisic acid (ABA), of rapeseed plants growth with amendment application showed in blue bars (AM) and non-treated plants showed in green bars (Control). Bars are means (*n* = 4) and capped lines are standard errors. Within each graph, bars with the different letter indicate that values are significantly different (*p* < 0.05) *t*-test.

To investigate the effect of organic amendment on rapeseed metabolism, a total of 306 proteins were analyzed. Comparison showed that 225 proteins increased in abundance, while 81 decreased significantly in amended plants (Table A1). Proteomic quantification showed protein accumulation in different cell organelles of rapeseed plants under amendment application. Among accumulated proteins, the chloroplast and nucleus had the highest proportions (23.5%), followed by the cytosol (13.6%). Additionally, the cell membrane and mitochondria showed percentages of accumulation of proteins of 8.6% and 5.6%, respectively (Figure 4A). Functional classification of proteins revealed strong upregulation of transmembrane and mitochondrial proteins involved in protein transport. Proteins mediating the metabolism of sugars and other metabolites such as glucose, malate, lipids, and ions also accumulated. Additionally, proteins related to transcription and protein folding were highly upregulated, suggesting enhanced protein biosynthesis. Proteins involved in the biosynthesis of key metabolites such as lignin, ABA, and sucrose also accumulated under amended conditions. Finally, proteins associated with stress responses, redox homeostasis, and plant energy status (e.g., aerobic respiration) were strongly upregulated (Figure 4B and Figure A1). On the other hand, some proteins related to the Calvin cycle, coenzyme A biosynthesis, photorespiration, and most of defense mechanisms and signaling proteins were found to be downregulated under amendment application. Other proteins mediating organelle organization, amino acid production, photosynthetic light reactions, and proteolysis could be found both upregulated and downregulated. Zeaxanthin epoxidase, a protein related to ABA biosynthesis, was also significantly upregulated under organic amendment application, showing increased levels of ABA (Figure 4B and Figure A1, Table A1). The upregulation of those mechanisms related to protein can be aligned with organic acid (quinic acid and nicotinic acid) accumulation possibly improving the redox homeostasis and stress signaling (Figure 3).

### 2.6. Soil Bacterial Analyses

**Figure 5:**
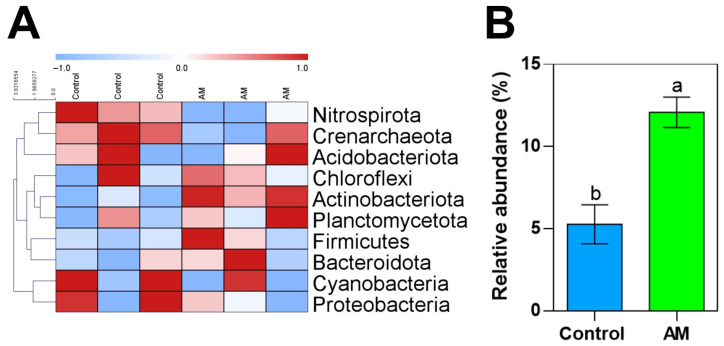
Heatmaps of relative abundance of identified bacterial phyla (**A**) and histogram of relative abundance (%) of Actinobacteriota phylum (**B**) of rhizosphere soil samples of rapeseed plants under control conditions (Control) and with amended plants (AM). Heatmap was arranged using Z = -score values, where colors ranging from red (increase) to green (decrease) are shown in the legend. Hierarchical clustering was arranged for identified phyla using Euclidean distance and average linkage. Histogram bars (**B**) are means (*n* = 3), and capped lines are standard errors. Within the graph, bars with different letters indicate that values are significantly different (*p* < 0.05) in *t*-test.

The relative abundance of different soil bacterial phyla was analyzed in rhizospheric samples from both rapeseed treatments. To assess the effect of the organic amendment on bacterial populations, taxonomic composition at the phylum level was compared using a heatmap. According to our results, bacterial phyla such as Nitrospirota and Crenarchaeota showed similar tendencies, being more relevant under control conditions, while other groups such as Chloroflexi, Actinobacteriota, and Planctomycetota tended to increase under amendment application. Statistical analyses showed that out of 10 represented phyla (Figure 5A and Table A2), only the Actinobacteriota phylum showed a significative increase under amendment application (Figure 5B). Similar levels of the Cyanobacteria phylum were found in both treatments. Actinobaterias include groups with important roles in soil nutrient cycling and organic matter degradation, so these changes could align with improving plant nutrition capabilities and plant responses to stresses.

## 3. Discussion

**Figure 6:**
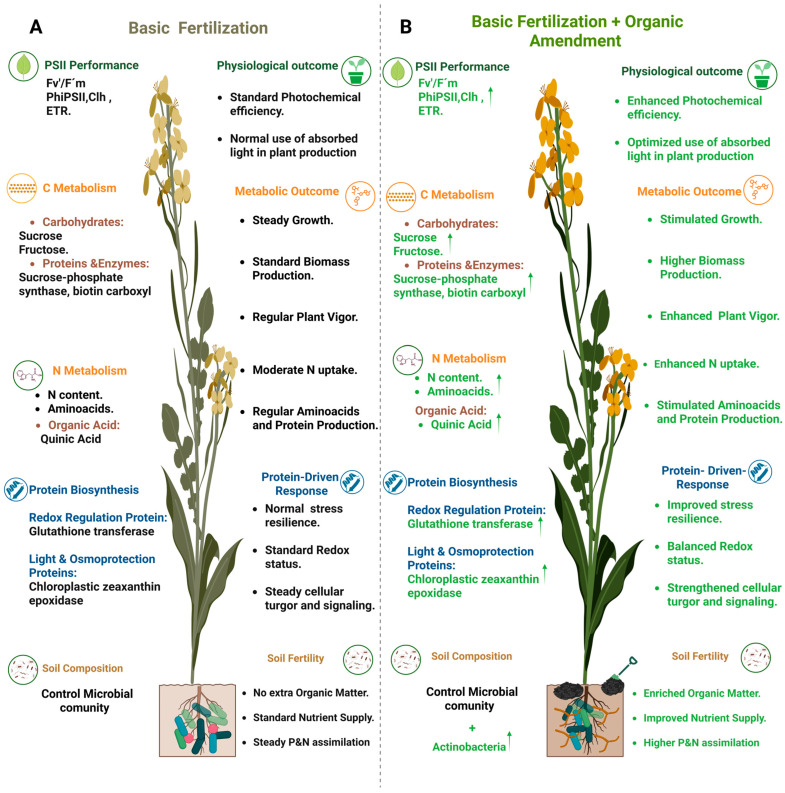
Conceptual model of rapeseed responses to (**A**) basic fertilization and (**B**) basic fertilization combined with organic amendment. The scheme integrates the major physiological, metabolic, protein-related, and soil-associated changes observed under each fertilization regime. Black text indicates responses under control/basic fertilization, while green text and arrows highlight enhancements induced by organic amendment.

As previously addressed, the Green Revolution sought to alleviate food scarcity using high-yielding crop varieties and chemical inputs. In this sense, organic manure plays a crucial role in soil health and plant nutrition. Previous studies [10,22] have shown that organic manures can improve crop productivity. Yet, these investigations have largely focused on agronomic outcomes, offering limited understanding of the underlying physiological and metabolic processes. Notably, the role of soil microbiota in shaping plant responses to organic amendments remains largely unexplored, a critical knowledge gap that the present study addresses. In this context, our study proposes a conceptual model (Figure 6) that illustrates the integrated responses when comparing basic fertilization alone and in combination with organic amendments. It was demonstrated that the addition of organic matter improved soil quality, leading to an enhanced nutrient availability and uptake. These changes activated key metabolic pathways, including carbon and nitrogen metabolism as well as specific protein-driven responses, ultimately strengthening stress resilience, boosting crop productivity, and improving overall plant physiology.

### 3.1. Amendment Application Contributes to Increase Leaf C and N Metabolism

The current study showed that, in agreement with previous studies, amendment application contributed to increased plant growth. Furthermore, we observed that the amendment-associated effects were consistent across leaves, shoots, and roots. Gas exchange analyses showed no significant differences in photosynthetic rates per unit leaf area between treatments; however, the 200% increase in leaf biomass in amended plants indicates a markedly greater canopy-level CO_2_ fixation capacity. Similar results were described by previous studies [23,24] where the leaf surface was the target factor conditioning plant growth under low-water-availability conditions. In this sense, the higher content of proteins related to growth promotion, such as 1,3-beta-glucan synthase (synthesizes important cell wall components) and xyloglucan endotransglucosylase/hydrolase (which is involved in cell wall remodeling), was also observed in fertilized plants.

Interestingly, chlorophyll fluorescence analyses revealed that rapeseed plants fertilized with amendment exhibited a significantly better photosynthetic efficiency and productivity in plants, which was reflected by the higher values in electron transport rate (ETR), maximum quantum yield of photosystem II (Fv’/Fm’), and effective quantum yield of photosystem II (PhiPS2). These results indicate that amendment application enhanced electron transport rates through the photosynthetic chain, improved the efficiency of converting absorbed light into chemical energy, and optimized photosystem II function, leading to an overall higher photosynthetic efficiency [25]. Proteomic analyses further confirmed this, revealing increased levels of chlorophyll and proteins associated with light harvesting (chlorophyll a-b binding protein, chloroplastic) and ADP/ATP transport (ADP/ATP carrier protein) in treated plants.

In line with such findings, the proteomic and metabolomic approaches confirmed that amendment-associated stimulation of leaf C metabolism was involved in the higher plant growth of those plants. Plants treated with the organic amendment had higher contents of carbohydrates (such as sucrose, fructose, D-glucose, etc.) and organic acids (such as quinic acid). The higher levels of carbohydrates and stimulation of the glycolysis pathway might have favored the generation of ATP and NADH, as stated by previous studies [26]. In line with this, the elevated presence of organic acids suggests a modulation of the tricarboxylic acid (TCA) cycle, a central pathway in cellular respiration. These organic acids serve as intermediates or products of the TCA cycle, participating in ATP production and the generation of metabolic intermediates crucial for plant growth and function. Those findings were supported by the proteome, where amendment application increased the content of proteins associated with C metabolism, such as sucrose-phosphate synthase, biotin carboxyl, and mitochondrial dihydroorotate dehydrogenase.

The plant nitrogen content is crucial for growth, as it supports the synthesis of proteins, enzymes, and other molecules essential for metabolic processes and overall development. The current study showed that in plants with amendment application, the leaf N content increased by 74%. The higher N content was reflected in an increase in amino acids’ contents, such as L-tryptophan. In this sense, the higher organic acid levels detected in plants treated with the organic amendment show an increased flux of organic acid-derived intermediates to sustain amino acid synthesis through the TCA cycle.

### 3.2. Improved Oxidative Stress Regulation Mechanisms

The ability to regulate oxidative stress is paramount for the survival and optimal functioning of living organisms, including plants. As was mentioned before, research investigations carried out with plants exposed to stressful conditions [13,14,27] have shown that the amendment plays a crucial role in significantly enhancing plant growth through the improvement of stress tolerance mechanisms. According to those studies, the application of organic fertilizers serves to mitigate reductions in chlorophyll levels, enhance photosynthetic processes, and diminish the accumulation of reactive oxygen species (ROS) as well as lipid peroxidation. These effects are critical for plant performance, as elevated ROS levels can lead to oxidative damage and impair physiological functions. Plants possess a complex antioxidant defense system, comprising enzymes (such as superoxide dismutase, catalase, and peroxidase), as well as non-enzymatic antioxidants like glutathione and ascorbate. Additionally, minor levels of anthocyanins indicate reduced abiotic stress, which is strongly correlated with ROS-generating stresses [28]. Therefore, investing strategies to enhance oxidative stress tolerance in plants is critical for ensuring crop resilience.

Proteomic analyses highlighted that amendment application increased the levels of relevant proteins (glutathione transferase, L-ascorbate peroxidase, and peroxidases) and metabolites (L-ascorbic acid and quinic acid) involved in the adjustment of redox homeostasis. Furthermore, this study also revealed a downregulation of glutathione hydrolase (involved in the breakdown of glutathione) content. Moreover, the build-up of another relevant protein associated with light management, such as chloroplastic zeaxanthin epoxidase, would confirm the better redox state adjustment of those plants. Osmoprotectants have also been described to play a crucial role in managing the redox status in plants through several mechanisms [29]. More specifically, their content has been associated with ROS scavenging (like proline) and other factors that might induce oxidative stress, such as the stabilization of proteins and cell membranes, cellular turgor maintenance, and signaling. In this sense, the current study revealed a higher content of osmoprotectants, such as trehalose, erythritol, and galactitol, in line with previous characterizations. Collectively, these functions help plants to better withstand stress and improve their capacity to adjust the redox status.

### 3.3. Impact of Organic Amendment in Soil Microbiota

Previous studies have highlighted the crucial role of organic amendments in improving soil quality by supplying essential carbon compounds that support plant growth, increasing the organic matter content, and promoting the proliferation of beneficial microorganisms. The utilization of organic amendments has been widely recognized for its significant contribution to enhancing disease resistance in plants, a phenomenon that is closely associated with alterations in the signaling pathways that regulate plant defense mechanisms. Furthermore, they serve to protect and enhance soil fertility, as underscored by prior research [22]. This is achieved through the stimulation of the soil’s microbial community and population, which is facilitated by the application of organic amendments, thus contributing to the overall health of both soil and plants. The application of organic amendments not only boosts the soil quality but also ensures sustainable agricultural practices by promoting a balanced soil ecosystem that supports plant growth and development. Our soil bacterial genomic analysis also revealed a significant increase in the Actinobacteriota phylum under amendment application. Actinobacteria are considered beneficial microorganisms because they enhance nutrient availability and solubilization, such as phosphate mobilization and nitrogen fixation. This taxonomic group also plays an important role in soil nutrient cycling and organic matter degradation [30]. In a context of a more sustainable agriculture with increasing demands, Actinobacteria soil enhancement and protection entails that organic amendment not only benefits plant nutrient assimilation but also strengthens plant–bacterial relationships related to nutrient cycling, in line with [31].

## 4. Materials and Methods

### 4.1. Plant Growth and Experimental Design

This investigation was carried out using the high-yielding Clearfield^®^ hybrid rapeseed (*Brassica napus* L.) cultivar Pioneer^®^ 44Y84. The pilot experiment was carried out in the Agrobiotechnology Institute (IdAB)-regulated greenhouse. The experimental period extended from the sowing of the plants to their harvest. The seeds were germinated in 7.5 L pots. After germination, pots were randomly distributed across the experimental space, with us establishing 1 plant per pot. The experiment employed a silty clay soil with a pH of 8.2, organic matter at 2.7%, total nitrogen at 1.6%, available phosphorus at 68 ppm, available potassium at 285 ppm, and cation exchange capacity of 13.3 meq 100 g^−1^. Basic fertilization was tailored to meet the crop’s needs based on this soil analysis. All plants were fertilized with ammonium sulfate at a rate of 300 L ha^−1^ (8% nitrogen, 21% sulfur trioxide, liquid; manufactured by Fertival©, Quintenic, France). Half of the plants (*n* = 4) served as controls, while the other half received the organic amendment Humival (manufactured by Fertival©, France), which was applied at a rate of 2000 kg ha^−1^ before sowing, with us mixing it with the soil homogenously. This amendment included 67% organic matter (from slurry, poultry, and a mixture of plant material), which provided 5% organic nitrogen, 5% phosphorus pentoxide, 2% potassium oxide, and 7% calcium oxide. The rapeseed seedlings were cultivated in a regulated greenhouse environment under natural sunlight conditions from May to August with an average photoperiod of 14–15 light hours, with temperatures maintained at 22/16 °C (day/night). Plants were irrigated at pot capacity, ensuring they were consistently kept at the maximum substrate water-holding capacity. Plants were not treated against any disease or pests.

At the flowering stage (BBCH 53), gas exchange and chlorophyll fluorescence measurements were performed, and leaf samples were collected. Samples were immediately frozen in liquid nitrogen and stored at –80 °C for subsequent analyses. Subsamples underwent oven drying for 48 h at 60 °C to facilitate further experimentation (described below). The final harvest was conducted when the plants reached the maturity stage (BBCH 89). At this stage, above-ground plant biomass was determined. Shoots (leaves and shoots) were harvested and later dried at 60 °C in an oven for 48 h to obtain the dry mass of each plant. The crop yield was also calculated as the seed DW (g) per plant.

### 4.2. N Content

Leaf nitrogen content was analyzed utilizing sample dynamic combustion with an elemental analyzer (FlashEA1112, ThermoFinnigan, Waltham, MA, USA) equipped with a MAS200R autosampler. The dried leaf samples were finely ground, and 1 mg was accurately weighed and stored in tin capsules for elemental analyses (MX5 microbalance, Mettler-Toledo, Columbus, OH, USA).

### 4.3. Gas Exchange and Chlorophyll Fluorescence

Fully expanded apical leaves were measured with a portable photosynthesis system (Li-Cor 6400) for gas exchange. The rate of CO_2_ assimilation under light saturation (An) was determined at a PPFD of 1200 μmol m^−2^ s^−1^ using established equations [32]. Stomatal conductance (g_s_) was quantified following the methodology outlined by [33]. The electron transport rate (ETR), maximal quantum efficiency of PSII (Fv/Fm), and the relative quantum efficiency of PSII photochemistry (ΦPSII) were simultaneously determined with a fluorescence chamber (LFC 6400-40; Li-COR) connected to the Li-Cor 6400XT portable photosynthesis system in their growth conditions at midday (10:00–13:00).

### 4.4. Chlorophyll and Anthocyanin Content Analysis

Chlorophyll (Chl) and anthocyanin (Anth) content were estimated using a portable non-destructive DUALEX sensor (Dualex Scientific, Force A, Orsay, France), measured before harvesting. Chlorophyll was measured in µg cm^−2^ in the range of 5–80 µg cm^−2^. Anthocyanins were measured using relative absorbance units from 0 to 1.5.

### 4.5. Proteomic Profile

Protein extraction was performed using 3 lyophilized leaf samples per treatment (*n* = 3), which were homogenized in a lysis buffer containing 5% sodium dodecyl sulfate (SDS) and 25 mM triethylammonium bicarbonate (TEAB). To reduce and alkylate the proteins, 5 mM tris(2-carboxyethyl)phosphine (TCEP) and 10 mM chloroacetamide (CAA) were added, followed by incubation at 60 °C for 30 min.

Homogenization was carried out using a micro-tip probe ultrasonicator (UP50H, Hielscher Ultrasonics, Teltow, Germany) for 1 min. The resulting homogenate was centrifuged at 16,000× *g* for 15 min at 4 °C, and the supernatant containing solubilized proteins was collected for downstream analysis. The samples were quantified using microBCA analysis (Pierce, Appleton, WI, USA), with equal amounts (5 µg per sample) being dissolved individually in a solution of 8 M urea and 25 mM ammonium bicarbonate. Subsequently, they were reduced with DTT and alkylated with iodoacetamide, following a methodology outlined by [34]. The digested samples were then mixed with 0.2% trifluoroacetic acid in water and analyzed using multiple reaction monitoring on a 1D Plus nanoLC Ultra system (Eksigent, Dublin, CA, USA) connected to a Sciex 5500 QTRAP triple quadrupole mass spectrometer (Sciex, Framingham, MA, USA) with a nano-electrospray ionization source and controlled by Analyst v.1.5.2 software (ABSciex, Marlborough, MA, USA). The tryptic digests were introduced online through a C18 PepMap, 300 µm internal diameter × 5 mm trapping column (5 µm, 100 Å, Thermo Scientific, Waltham, MA, USA), and separated using a BioSphere C18, 75 µm internal diameter × 150 mm capillary column (3 µm, 120 Å, angstroms). A set of 84 transitions (typically 3–4 per peptide, with a preference for higher-mass y series ions) for 21 distinct peptides chosen from 10 different proteins was under observation. The software Skyline (Version 24.1) automatically established collision energy values for the specified peptides based on the methodology outlined by [35]. Protein quantification was performed by calculating protein ratios based on their measured abundances. Statistical significance of differential expression was assessed using a background-based *t*-test (*n* = 3).

For quantitation, only protein groups (master proteins) with a False Discovery Rate (FDR) < 1% and with abundance values in both standards (IS) were included. To identify differentially expressed proteins in each comparison, a Benjamini–Hochberg correction was applied to control for multiple testing. Proteins with an adjusted *p*-value ≤ 0.05 were considered significantly differentially expressed.

To determine the functional characteristics and subcellular localization of the identified proteins, their sequences were mapped to the UniProtKB/Swiss-Prot database (https://www.uniprot.org/; accessed on 23 March 2023). To calculate the *p*-values for quantification results, the *t*-test (background-based) statistical method was used. Those proteins differentially expressed with an adjusted *p*-value ≥ 0.05 were considered significant.

### 4.6. Gas Chromatography–Mass Spectrometry (GC-MS) Analyses

The process of metabolite extraction via gas chromatography–mass spectrometry (GC-MS) following the next protocol aligned with GC-MS-Based Untargeted Metabolomics: 6 mg of 3 lyophilized leaves per each treatment (*n* = 3) was mixed with a 1 mL solution of H_2_O/ACN/isopropanol (2/3/3) containing 4 mg L^−1^ of ribitol. After centrifugation, 70 µL of myristic acid-d27 in H_2_O/MeOH/isopropanol (2/5/2) at a concentration of 0.3 g L^−1^ were added to the supernatant. These samples were then vacuum spin-dried and stored at −80 °C. Methoxyamine, dissolved in pyridine at a concentration of 20 mg L^−1^, was utilized to dissolve the samples, with a 90 min incubation at 30 °C, continuously shaking. N-methyl-N-trimethylsilyl-trifluoroacetamide (MSTFA, 80 µL) was then added and incubated for 30 min. The derivatization mix afterwards underwent 2 h of incubation at room temperature (RT). Prior to GC autosampler loading, a series of eight alkanes (C10 to C36) was incorporated. Analysis involved injecting 1 μL in splitless mode at an injector temperature of 230 °C. Chromatographic separation was performed using helium as the gas carrier at a flow rate of 1 mL min^−1^ in constant flow mode, with a temperature ramp from 80 to 330 °C over 2 to 18 min, followed by 6 min at 330 °C. Ionization was carried out via electron impact at 70 keV, with an MS acquisition rate of 20 spectra s^−1^ across the *m*/*z* range 80–500. Peak identification was performed by comparing the fragmentation pattern with MS databases (NIST) using a match cut-off criterion of 700/1000, and based on the retention index (RI) with the alkane series as retention standards. Peak integration was executed using the LECO Pegasus software, with manual confirmation for each compound in all analyses due to sporadic automated integration errors.

A collection of 84 transitions (typically 3–4 per peptide, with a preference for higher-mass y series ions) for 21 distinct peptides chosen from 10 different proteins was monitored. The software Skyline (Version 24.1) automatically established collision energy values for the specified peptides based on the methodology outlined by [35].

### 4.7. Genomic Analyses of Soil Bacteria

DNA from rhizospheric soil samples was extracted using the DNeasy PowerSoil Kit (Qiagen, Germany; REF21802, LOT ZQ031) following the protocol from the manufacturer. The composition and structure of the sampled bacterial communities from the samples were assessed through the amplification and sequencing the V3-V4 variable regions of the 16S rRNA gene using primers (515f/806r) that incorporate Illumina adaptor sequences and indexing barcodes [36]. The Illumina Miseq sequencing 300 × 2 approach was used for both communities. Amplification was performed after 25 PCR cycles. A negative control of the DNA extraction was included as well as a positive Mock Community control to ensure quality control. Bioinformatics processing and analysis of raw demultiplexed forward and reverse reads were processed as shown in the following table using QIIME2 [37]. Step methods for bioinformatic analyses were performed according to Dada2 [38]. Phylogeny assessment was performed according to MAFFT method [39] and FastTree [40] method. Phylotype data was used to calculate observed OTUs (community richness). Taxonomic assignment of phylotypes was performed using a Bayesian classifier trained with Silva database version 138 (99% OTU full-length sequences) [41]. Relative abundance was obtained by dividing the assigned phylotypes for each taxonomic group by the total population.

### 4.8. ABA Quantification

We suspended 40 mg of lyophilized leaf sample in 80% methanol–1% acetic acid containing internal standards and mixed by shaking for 1 h at 4 °C. The extract was kept at −20 °C overnight and then centrifuged and the supernatant dried in a vacuum evaporator. The dry residue was dissolved in 1% acetic acid and passed through an Oasis HLB (reverse phase) column as described by [42]. For abscisic acid (ABA) quantification, the dried eluate was dissolved in 5% acetonitrile–1% acetic acid, and the hormone was separated using an autosampler and reverse-phase UHPLC chromatography (2.6 µm Accucore RP-MS column, 100 mm length, 2.1 mm diameter; ThermoFisher Scientific, Waltham, MA, USA) with a 5 to 50% acetonitrile gradient containing 0.05% acetic acid, at 400 µL/min over 21 min.

### 4.9. Statistical Analysis

Control plants and amended plants were statistically analyzed by T-student (*t*-test) using GraphPad PRISMV6.0 (GraphPad Software). Differences were significant when a *p*-value < 0.05 was used to determine the significance between experimental groups. Heatmaps for metabolite and soil bacteria analyses were arranged using Z-score values via the RStudio software (Version 2024.09.0+375). Hierarchical clustering was arranged for identified metabolites and soil bacterial phyla using the Euclidean distance and average linkage, with a *p*-value threshold < 0.05.

## 5. Conclusions

The results of this study demonstrate that organic amendment positively modulates multiple metabolic mechanisms in rapeseed, enhancing the redox balance and overall homeostasis. Treated plants exhibited more efficient light energy utilization and activation of pathways involved in antioxidant synthesis and metabolism, which are critical for coping with biotic and abiotic stresses. Moreover, amendment application increased the abundance of soil Actinobacteria, contributing to improved soil quality and supporting plant nutrient assimilation and stress resilience. Together, these responses indicate that organic amendments can enhance plants’ capacity to withstand oxidative stress and maintain metabolic homeostasis under changing environmental conditions. This study provides new insights into the physiological and metabolic processes by which organic amendments improve plant productivity and resilience, highlighting their potential role in sustainable agricultural management. However, possible limitations of this study could be discussed, and further research is still needed to better understand the effect of organic amendments on physiological responses of rapeseed.

## Figures and Tables

**Table 1:** Biomass, shoot weight (SW), leaf weight (LW), root weight (RW), grain yield, and leaf nitrogen content (N) of rapeseed plants growth with amendment application (AM) and non-treated plants (Control). Values are means ± S.E. (*n* = 4), values with different letters indicate that values are significantly different (*p* < 0.05) in *t*-test.

Treatment	Biomass (g plant^−1^)	SW (g plant^−1^)	LW (g plant^−1^)	RW (g plant^−1^)	Yield (g plant^−1^)	N (%)
Control	10.7 ± 0.52 b	9.84 ± 0.63 b	0.83 ± 0.27 b	4.24 ± 1.05 a	276 ± 25.2 a	2.3 ± 0.40 b
AM	19.4 ± 0.83 a	16.4 ± 0.26 a	3.01 ± 0.62 a	7.34 ± 1.55 a	748 ± 245 a	4.0 ± 0.24 a

## Data Availability

The original contributions presented in this study are supported in the article; further inquiries can be directed to the corresponding authors.

## Abbreviations

The following abbreviations are used in this manuscript:

A_n_	Net photosynthetic CO_2_ assimilation
g_s_	Stomatal conductance
C_i_	Sub-stomatal CO_2_ concentration
E	Transpiration rate
PPFD	Photosynthetic photon flux density
ETR	Electron transport rate
Fv’/Fm’	Intrinsic quantum yield of PSII photochemistry
PhiPS2	Photosystem II efficiency
WUE	Water use efficiency
Chl	Chlorophyll
Anth	Anthocyanin
ROS	Reactive oxygen species
TCA	Tricarboxylic acid
ppm	Part per million
CO_2_	Carbon dioxide
ICP/OES	Inductively coupled plasma/optical emission spectrometry
N	Nitrogen
C	Carbon
ATP	Adenosine triphosphate
ADP	Adenosine diphosphate
DNA	Deoxyribonucleic acid
OTUs	Observed operational taxonomic units
AM, Amen	Organic amended
*t*-test	Student’s *t*-test

## Appendix A

**Table A1:** *List of differentialy accumulated proteins identified in oilseed rape plants in response to organic amendment fertilization*.

Accession	Protein Name	Unique Peptides	Localization	Function	Abundance Ratio	Abundance Ratio (log2)	*p*-Value
A0A078H9F9	Reticulon-like protein	1	Endoplasmic reticulum	ER tubular network organization	3.06	1.61	2.64 × 10^−15^
A0A078GHF0	Reticulon-like protein	3	Endoplasmic reticulum	ER tubular network organization	2.547	1.35	2.64 × 10^−15^
A0A078FBI2	BnaCnng02600D protein	3	-	Response to stress	2.403	1.27	2.64 × 10^−15^
A0A078IVR4	FACT complex subunit	1	Nucleus	DNA repair and replication	2.414	1.27	2.17 × 10^−12^
A0A078I4R4	Reticulon-like protein	3	Endoplasmic reticulum	ER tubular network organization	2.318	1.21	2.64 × 10^−15^
A0A078GVS3	Imidazole glycerol phosphate synthase hisHF	1	Chloroplast	Amino acid metabolism	2.272	1.18	3.57 × 10^−11^
A0A078IXL1	BnaC08g47240D protein	1	Nucleus	Transcription	2.185	1.13	1.19 × 10^−10^
A0A078ILU3	(rape) hypothetical protein	1	-	Defense response	2.12	1.08	8.22 × 10^−10^
A0A078FR89	BnaA01g34270D protein	3	Mitochondria	Aerobic respiration	2.096	1.07	2.64 × 10^−15^
A0A078ICU7	(rape) hypothetical protein	3	Nucleus	Transcription	2.067	1.05	2.64 × 10^−15^
A0A078IB52	(rape) hypothetical protein	3	-	Unknown	2.072	1.05	2.64 × 10^−15^
A0A078IZU4	(rape) hypothetical protein	3	-	Protein folding	2.055	1.04	2.64 × 10^−15^
A0A078G2L0	NAD(P)H-quinone oxidoreductase subunit 5	6	Chloroplast	Photosynthetic light reactions	1.981	0.99	2.64 × 10^−15^
A0A078G3V0	BnaA09g01870D protein	1	Vacuole	Protein transport	1.975	0.98	2.78 × 10^−12^
A0A078J4S6	BnaA09g54590D protein	1	Nucleus	Transcription	1.976	0.98	1.51 × 10^−9^
A0A078IQJ7	(rape) hypothetical protein	1	-	Protein folding	1.964	0.97	2.64 × 10^−15^
A0A078I462	(rape) hypothetical protein	2	-	Protein folding	1.932	0.95	2.64 × 10^−15^
A0A078FPS6	(rape) hypothetical protein	3	-	Unknown	1.938	0.95	7.82 × 10^−12^
A0A078GHF6	(rape) hypothetical protein	2	Nucleus	Transcription	1.889	0.92	2.64 × 10^−15^
A0A078FU32	BnaA08g02930D protein	3	Nucleus	Transcription	1.882	0.91	2.64 × 10^−15^
A0A078G143	(rape) hypothetical protein	1	Chloroplast	Amino acid metabolism	1.852	0.89	2.64 × 10^−15^
A0A078G4P9	BnaC09g29190D protein	6	-	Aerobic respiration	1.816	0.86	2.64 × 10^−15^
A0A078FCG8	(rape) hypothetical protein	15	Cell membrane	Ion transport	1.818	0.86	2.64 × 10^−15^
A0A078J890	(rape) hypothetical protein	8	Nucleus	Unknown	1.773	0.83	2.64 × 10^−15^
A0A078JLP0	(rape) hypothetical protein	13	Cytosol	Response to stress	1.766	0.82	2.64 × 10^−15^
A0A078HZ14	BnaC08g10640D protein	8	-	Protein transport	1.737	0.8	2.64 × 10^−15^
A0A078H4V5	(rape) hypothetical protein	5	Cytosol	Response to stress	1.713	0.78	2.64 × 10^−15^
A0A078I5S5	(rape) hypothetical protein	1	-	Response to stress	1.715	0.78	3.20 × 10^−5^
A0A078H252	(rape) hypothetical protein	4	Nucleus	Chromatin organization	1.696	0.76	2.64 × 10^−15^
A0A078GEQ7	BnaC04g01660D protein	1	-	Unknown	1.679	0.75	5.21 × 10^−4^
A0A078EZB5	(rape) hypothetical protein	1	-	Unknown	1.668	0.74	4.50 × 10^−9^
A0A078FNQ6	BnaC08g40680D protein	2	-	Unknown	1.664	0.73	2.44 × 10^−5^
Q69BP4	(rape) hypothetical protein	13	Cytosol	Response to stress	1.646	0.72	2.64 × 10^−15^
A0A078IXP0	(rape) hypothetical protein	3	-	Amino acid metabolism	1.653	0.72	2.25 × 10^−12^
A0A078JHC6	BnaCnng49050D protein	1	Nucleus	Chromatin organization	1.649	0.72	2.34 × 10^−8^
A0A078J5L0	(rape) hypothetical protein	1	Cytosol	Unknown	1.635	0.71	1.52 × 10^−4^
A0A078IX55	(rape) hypothetical protein	4	Chloroplast	Malate transport	1.625	0.7	2.64 × 10^−15^
A0A078FBT9	(rape) hypothetical protein	3	-	Unknown	1.625	0.7	2.97 × 10^−9^
A0A078H492	BnaA06g36180D protein	5	Chloroplast	Unknown	1.615	0.69	2.64 × 10^−15^
A0A078FIZ4	CDP-diacylglycerol--inositol 3-phosphatidyltransferase	2	Golgi apparatus	Phospholipid metabolism	1.614	0.69	2.94 × 10^−5^
A0A078F8Q4	(rape) hypothetical protein	2	Nucleus	Transcription	1.601	0.68	5.55 × 10^−14^
A0A078I5B1	(rape) hypothetical protein	2	Endoplasmic reticulum	Lipid metabolism	1.606	0.68	6.55 × 10^−11^
A0A078JJ05	(rape) hypothetical protein	1	-	Unknown	1.603	0.68	6.55 × 10^−11^
A0A078JNA0	BnaCnng57110D protein (Fragment)	1	Cell membrane	Ion transport	1.603	0.68	8.62 × 10^−4^
A0A078G3S7	BnaC09g29130D protein	25	Chloroplast	Protein transport	1.595	0.67	2.64 × 10^−15^
A0A078FUQ6	(rape) hypothetical protein	9	Nucleus	Chromatin organization	1.583	0.66	2.64 × 10^−15^
A0A078G765	BnaC02g28790D protein	2	Nucleus	Transcription	1.584	0.66	4.93 × 10^−8^
A0A078FQZ2	(rape) hypothetical protein	2	Chloroplast	Unknown	1.575	0.66	2.96 × 10^−7^
A0A078H3B2	(rape) hypothetical protein	1	Chloroplast	Malate transport	1.582	0.66	1.42 × 10^−5^
A0A078FCS9	BnaC06g23930D protein	1	-	Response to stress	1.556	0.64	7.04 × 10^−7^
A0A078IBS7	(rape) hypothetical protein	5	-	Unknown	1.553	0.64	8.37 × 10^−7^
A0A078FLH2	(rape) hypothetical protein	1	-	Lipid metabolism	1.559	0.64	1.75 × 10^−3^
A0A078I8F7	(rape) hypothetical protein	3	-	Response to stress	1.551	0.63	1.07 × 10^−13^
A0A078I275	BnaC08g04910D protein	1	-	Lipid metabolism	1.543	0.63	2.59 × 10^−13^
A0A078HYY2	(rape) hypothetical protein	3	Chloroplast	Malate transport	1.548	0.63	2.59 × 10^−13^
A0A078HNK9	Protein-serine/threonine phosphatase	3	-	Signaling	1.543	0.63	3.46 × 10^−10^
A0A078FGL5	(rape) hypothetical protein	3	Endoplasmic reticulum	Protein transport	1.551	0.63	3.39 × 10^−6^
A0A078HQG5	(rape) hypothetical protein	4	Nucleus	Transcription	1.534	0.62	1.71 × 10^−8^
A0A078J925	BnaC07g48010D protein	3	Apoplast	Unknown	1.537	0.62	2.62 × 10^−8^
A0A078H561	BnaA10g12120D protein	7	-	Glucose transport	1.525	0.61	1.30 × 10^−12^
A0A078GMS3	(rape) hypothetical protein	4	-	Carbohydrate metabolism	1.522	0.61	3.08 × 10^−6^
A0A078HE96	BnaC02g38300D protein	3	Cell membrane	Unknown	1.516	0.6	5.67 × 10^−6^
A0A078GWK5	BnaC07g32090D protein	1	Chloroplast	Unknown	1.513	0.6	3.11 × 10^−5^
A0A078HCV4	(rape) hypothetical protein	1	Cell membrane	Ion transport	1.514	0.6	5.85 × 10^−3^
A0A078GAB1	BnaC09g00530D protein	1	-	Signaling	1.513	0.6	7.06 × 10^−3^
A0A078GHL0	(rape) hypothetical protein	1	Vacuole	Ion transport	1.493	0.58	1.61 × 10^−10^
A0A078G686	BnaC09g00810D protein	2	Vacuole	Ion transport	1.498	0.58	2.05 × 10^−4^
A0A078GKT1	(rape) hypothetical protein	1	Vacuole	Ion transport	1.494	0.58	2.06 × 10^−4^
A0A078IXK4	Potassium transporter	3	Cell membrane	Ion transport	1.493	0.58	1.09 × 10^−3^
A0A078FPE7	(rape) hypothetical protein	10	Vacuole	Unknown	1.489	0.57	2.85 × 10^−11^
A0A078JC11	BnaCnng45920D protein	3	Nucleus	Unknown	1.481	0.57	5.48 × 10^−8^
A0A078GWJ7	BnaA06g14580D protein	1	Nucleus	Chromatin organization	1.475	0.56	2.08 × 10^−5^
A0A078F9L1	BnaA05g27040D protein	1	-	Unknown	1.478	0.56	2.40 × 10^−5^
A0A078JHT5	(rape) hypothetical protein	3	Nucleus	Transcription	1.475	0.56	2.48 × 10^−5^
A0A078IXW2	(rape) hypothetical protein	2	-	Unknown	1.476	0.56	6.58 × 10^−5^
A0A078FI66	(rape) hypothetical protein	5	-	Unknown	1.469	0.55	1.60 × 10^−10^
A0A078JGM8	(rape) hypothetical protein	2	Vacuole	Sugar transport	1.467	0.55	2.05 × 10^−8^
A0A078HFX4	BnaC02g31330D protein	1	Mitochondria	Aerobic respiration	1.468	0.55	2.05 × 10^−4^
F8K8N9	(rape) hypothetical protein	1	Mitochondria	Ribosome biogenesis	1.464	0.55	2.43 × 10^−2^
A0A078HDB7	BnaC02g09720D protein	4	-	Unknown	1.449	0.54	2.23 × 10^−9^
A0A078JBL3	BnaC03g78270D protein	6	-	Response to stress	1.454	0.54	3.09 × 10^−8^
A0A078G8E9	(rape) hypothetical protein	10	Cell membrane	Ion transport	1.452	0.54	3.78 × 10^−8^
A0A078F7I4	(rape) hypothetical protein	2	-	Unknown	1.455	0.54	1.05 × 10^−4^
A0A078H8B0	(rape) hypothetical protein	1	Nucleus	Transcription	1.452	0.54	2.35 × 10^−4^
A0A078I8I3	(rape) hypothetical protein	4	-	Ion transport	1.44	0.53	7.31 × 10^−8^
A0A078JPD9	Thioglucosidase (Fragment)	2	Vacuole	Glucosinolate metabollism	1.444	0.53	3.50 × 10^−7^
A0A078FS08	(rape) hypothetical protein	1	-	Unknown	1.448	0.53	6.46 × 10^−3^
A0A078FHQ0	BnaC09g38510D protein	1	-	DNA repair and replication	1.449	0.53	1.93 × 10^−2^
A0A078F8K3	BnaA02g26510D protein	4	Nucleus	Proteolysis	1.436	0.52	2.23 × 10^−9^
A0A078J441	BnaA09g52620D protein	5	-	Transcription	1.43	0.52	1.46 × 10^−6^
A0A078GNT1	Thioglucosidase	8	Vacuole	Glucosinolate metabollism	1.423	0.51	6.03 × 10^−9^
A0A078F5Z9	BnaA05g27150D protein	5	Chloroplast	Unknown	1.422	0.51	8.02 × 10^−8^
A0A078GQ28	(rape) hypothetical protein	6	Nucleus	Chromatin organization	1.428	0.51	1.40 × 10^−6^
A0A078JC75	BnaAnng17860D protein	3	-	Unknown	1.42	0.51	3.65 × 10^−4^
A0A078FS29	(rape) hypothetical protein	2	-	Unknown	1.423	0.51	1.75 × 10^−3^
A0A078FTF1	BnaA08g28520D protein	1	-	Unknown	1.423	0.51	9.96 × 10^−3^
A0A078J084	(rape) hypothetical protein	2	-	Unknown	1.429	0.51	1.83 × 10^−2^
A0A078HV25	BnaC04g00810D protein	4	Nucleus	Transcription	1.416	0.5	1.59 × 10^−4^
A0A078DH63	(rape) hypothetical protein	2	Endosome	Vesicle mediated transport	1.41	0.5	2.86 × 10^−4^
A0A078IVG4	(rape) hypothetical protein	2	-	Unknown	1.407	0.49	4.72 × 10^−6^
A0A078FPB0	Non-specific lipid-transfer protein	2	-	Lipid transport	1.402	0.49	5.61 × 10^−6^
A0A078FTJ8	Chloride channel protein	4	-	Ion transport	1.405	0.49	5.61 × 10^−6^
A0A078FQK8	(rape) hypothetical protein	4	-	Unknown	1.398	0.48	3.91 × 10^−8^
A0A078HHL6	Thioglucosidase	13	Vacuole	Glucosinolate metabollism	1.397	0.48	4.35 × 10^−8^
A0A078G2E4	BnaC05g13830D protein	8	-	Unknown	1.395	0.48	6.17 × 10^−8^
A0A078IY82	(rape) hypothetical protein	5	-	Response to stress	1.392	0.48	8.37 × 10^−7^
A0A078IZ88	(rape) hypothetical protein	2	Nucleus	Transcription	1.39	0.48	1.92 × 10^−6^
A0A078IJX2	(rape) hypothetical protein	4	-	Unknown	1.393	0.48	3.16 × 10^−6^
A0A078ITH8	BnaA09g15670D protein	1	-	Unknown	1.398	0.48	4.46 × 10^−3^
A0A078I5M0	BnaA06g34100D protein	1	-	Proteolysis	1.391	0.48	2.36 × 10^−2^
A0A078G4P5	(rape) hypothetical protein	10	Chloroplast	Protein transport	1.382	0.47	8.88 × 10^−7^
A0A078HST0	BnaC05g43420D protein	1	-	Translation	1.387	0.47	3.40 × 10^−3^
A0A078IF26	1,3-beta-glucan synthase	4	Cell membrane	Cell wall organization	1.373	0.46	4.68 × 10^−4^
A0A078FZE9	BnaA06g35320D protein	13	Nucleus	DNA repair and replication	1.367	0.45	2.52 × 10^−7^
A0A078IID2	BnaA02g19360D protein	6	-	Transcription	1.368	0.45	3.93 × 10^−5^
A0A078I4Y5	Germin-like protein	4	Apoplast	Unknown	1.371	0.45	9.36 × 10^−4^
A0A078GN58	BnaA06g13580D protein	2	Vacuole	Sugar transport	1.365	0.45	1.49 × 10^−3^
A0A078HR75	(rape) hypothetical protein	3	Chloroplast	Unknown	1.368	0.45	1.75 × 10^−3^
A0A078GQ62	V-type proton ATPase subunit G	1	Vacuole	Ion transport	1.365	0.45	2.56 × 10^−2^
A0A078JMC4	BnaA09g54410D protein	5	-	Unknown	1.36	0.44	2.52 × 10^−7^
A0A078G5T7	(rape) hypothetical protein	13	Nucleus	Chromatin organization	1.359	0.44	7.62 × 10^−7^
A0A078FZ88	T-complex protein 1 subunit delta	1	Cytosol	Protein folding	1.356	0.44	2.00 × 10^−4^
A0A078GQS7	(rape) hypothetical protein	2	-	Unknown	1.359	0.44	9.40 × 10^−3^
A0A078HF75	(rape) hypothetical protein	1	Nucleus	Unknown	1.352	0.44	1.50 × 10^−2^
A0A078HWR7	Endoplasmic reticulum transmembrane protein	1	Endoplasmic reticulum	Protein transport	1.359	0.44	3.88 × 10^−2^
A0A078FU98	Copper transport protein	1	Cell membrane	Ion transport	1.343	0.43	3.18 × 10^−3^
A0A078IX79	(rape) hypothetical protein	1	-	Unknown	1.346	0.43	9.86 × 10^−3^
A0A078HX16	L-ascorbate peroxidase	2	Chloroplast	Redox homeostasis	1.348	0.43	1.49 × 10^−2^
A0A078FKD7	BnaA03g51800D protein	6	Nucleus	DNA repair and replication	1.337	0.42	1.08 × 10^−5^
A0A078ID05	BnaA02g24310D protein	3	-	Unknown	1.34	0.42	4.34 × 10^−5^
A0A078G149	(rape) hypothetical protein	2	-	Lipid metabolism	1.339	0.42	4.14 × 10^−3^
A0A078HUF2	Reticulon-like protein	3	Endoplasmic reticulum	Unknown	1.339	0.42	2.21 × 10^−2^
A0A078IPP8	Glutathione transferase	3	Cytosol	Redox homeostasis	1.326	0.41	1.44 × 10^−4^
A0A078I263	Chlorophyll a-b binding protein	2	Chloroplast	Photosynthetic light reactions	1.329	0.41	3.56 × 10^−4^
A0A078GI82	Thioglucosidase	2	-	Glucosinolate metabollism	1.325	0.41	6.70 × 10^−3^
A0A078IZG5	BnaA06g38150D protein	2	-	Ion transport	1.325	0.41	1.07 × 10^−2^
A0A078IBE6	BnaCnng16060D protein	1	Vacuole	Ion transport	1.324	0.41	2.32 × 10^−2^
A0A078G1M4	BnaC04g05890D protein	1	Nucleus	Unknown	1.325	0.41	2.62 × 10^−2^
A0A078ICP6	(rape) hypothetical protein	3	-	Unknown	1.316	0.4	2.05 × 10^−5^
A0A078JP84	BnaCnng59680D protein (Fragment)	9	-	Unknown	1.316	0.4	5.80 × 10^−5^
A0A078G9H2	(rape) hypothetical protein	2	-	Response to stress	1.319	0.4	1.08 × 10^−2^
A0A078FDJ2	(rape) hypothetical protein	2	Cytosol	Unknown	1.315	0.4	4.08 × 10^−2^
A0A078JIV5	(rape) hypothetical protein	6	Cell membrane	Response to stress	1.311	0.39	1.85 × 10^−5^
A0A078GU87	Carbonic anhydrase	7	-	Inorganic carbon utilization	1.312	0.39	2.00 × 10^−5^
A0A078J666	(rape) hypothetical protein	3	-	Unknown	1.314	0.39	1.20 × 10^−4^
A0A078IZX1	BnaA02g20200D protein	2	-	Unknown	1.313	0.39	1.59 × 10^−4^
A0A078JI91	(rape) hypothetical protein	2	-	Unknown	1.315	0.39	7.85 × 10^−3^
A0A078GJA8	BnaA05g30820D protein	3	Golgi apparatus	Cell wall organization	1.306	0.39	2.16 × 10^−2^
A0A078IGY8	Allene-oxide cyclase	3	Chloroplast	Jasmonic acid metabolism	1.305	0.38	4.49 × 10^−3^
A0A078HP42	(rape) hypothetical protein	2	Chloroplast	Unknown	1.305	0.38	1.13 × 10^−2^
A0A078HSH2	(rape) hypothetical protein	3	-	Unknown	1.293	0.37	3.11 × 10^−3^
A0A078G6G4	Dihydrolipoyllysine-residue succinyltransferase	1	Mitochondria	TCA cycle	1.296	0.37	4.83 × 10^−2^
A0A078FAM6	ABC-type xenobiotic transporter	19	-	Unknown	1.28	0.36	1.57 × 10^−4^
A0A078IWZ8	Transmembrane 9 superfamily member	1	Golgi apparatus	Protein transport	1.285	0.36	6.96 × 10^−4^
A0A078GN01	(rape) hypothetical protein	1	Vacuole	Ion transport	1.287	0.36	1.61 × 10^−2^
A0A078GHK2	BnaA06g00960D protein	3	-	Unknown	1.283	0.36	1.72 × 10^−2^
A0A078G4K4	Co-chaperone protein p23	4	Nucleus	Protein folding	1.288	0.36	2.09 × 10^−2^
Q9ZSL7	Non-specific lipid-transfer protein	2	-	Lipid transport	1.274	0.35	2.73 × 10^−4^
A0A078I6I1	BnaCnng12500D protein	1	-	Unknown	1.275	0.35	2.36 × 10^−2^
A0A078I056	(rape) hypothetical protein	3	-	Unknown	1.273	0.35	2.97 × 10^−2^
A0A078JND8	(rape) hypothetical protein	1	Cytosol	Redox homeostasis	1.278	0.35	3.14 × 10^−2^
A0A078J2 × 0	BnaA07g38390D protein	2	-	Unknown	1.27	0.35	3.58 × 10^−2^
A0A078J4Y4	ADP, ATP carrier protein	13	Chloroplast	ATP transport	1.262	0.34	4.40 × 10^−4^
A0A078GX94	(rape) hypothetical protein	5	-	Unknown	1.263	0.34	6.25 × 10^−4^
A0A078HK20	Non-specific lipid-transfer protein	2	-	Lipid transport	1.263	0.34	1.67 × 10^−3^
A0A078FC69	BnaA03g46100D protein	4	-	Unknown	1.267	0.34	3.43 × 10^−3^
A0A078JJB9	(rape) hypothetical protein	1	-	Unknown	1.262	0.34	4.54 × 10^−2^
A0A078IVF6	(rape) hypothetical protein	4	Mitochondria	Translation	1.261	0.34	4.87 × 10^−2^
Q84 × 96	3-oxoacyl-[acyl-carrier-protein] reductase	8	Chloroplast	Lipid metabolism	1.253	0.33	1.19 × 10^−3^
A0A078FIN1	(rape) hypothetical protein	7	Vacuole	Ion transport	1.254	0.33	2.95 × 10^−3^
A0A078HLQ1	(rape) hypothetical protein	2	-	Unknown	1.254	0.33	2.18 × 10^−2^
A0A078GE42	(rape) hypothetical protein	3	Nucleus	Transcription	1.256	0.33	2.84 × 10^−2^
A0A078HAX8	BnaA05g16460D protein	4	-	Unknown	1.251	0.32	7.37 × 10^−4^
A0A078H280	(rape) hypothetical protein	7	Vacuole	Unknown	1.249	0.32	3.26 × 10^−3^
A0A078GNY7	(rape) hypothetical protein	3	-	Unknown	1.25	0.32	4.48 × 10^−3^
A0A078J719	(rape) hypothetical protein	4	-	Unknown	1.248	0.32	4.98 × 10^−3^
A0A078GZX0	(rape) hypothetical protein	15	Nucleus	Chromatin organization	1.243	0.31	1.31 × 10^−3^
A0A078I5D5	Sucrose-phosphate synthase	8	-	Sucrose biosynthesis	1.242	0.31	1.40 × 10^−3^
A0A078G056	(rape) hypothetical protein	4	Chloroplast	Unknown	1.238	0.31	3.48 × 10^−3^
A0A078FZM8	Zeaxanthin epoxidase	5	Chloroplast	ABA biosynthesis	1.24	0.31	4.24 × 10^−2^
A0A078J1V7	(rape) hypothetical protein	6	Cytosol	Unknown	1.23	0.3	2.81 × 10^−3^
A0A078GPH3	Eukaryotic translation initiation factor 3 subunit C	1	Cytosol	Translation	1.229	0.3	2.83 × 10^−3^
A0A078FYW6	Non-specific lipid-transfer protein	1	-	Lipid transport	1.232	0.3	8.50 × 10^−3^
A0A078I7K6	Chalcone-flavonone isomerase family protein	5	Chloroplast	Lipid metabolism	1.235	0.3	1.75 × 10^−2^
A0A078INI4	BnaC01g44250D protein	4	-	Amino acid metabolism	1.232	0.3	1.92 × 10^−2^
A0A078HHQ4	(rape) hypothetical protein	5	Nucleus	Chromatin organization	1.229	0.3	2.16 × 10^−2^
A0A078HPA3	(rape) hypothetical protein	15	-	Unknown	1.221	0.29	4.32 × 10^−3^
A0A078JG83	(rape) hypothetical protein	7	-	Unknown	1.226	0.29	4.42 × 10^−3^
A0A078J0C6	BnaC07g50320D protein	4	-	Unknown	1.226	0.29	4.46 × 10^−3^
A0A078FUY5	Dihydroorotate dehydrogenase (quinone)	9	Mitochondria	Pyrimidine biosynthesis	1.225	0.29	4.62 × 10^−3^
A0A078I3D0	(rape) hypothetical protein	6	-	Unknown	1.221	0.29	8.26 × 10^−3^
A0A078G3T1	Chlorophyll a-b binding protein	2	Chloroplast	Photosynthetic light reactions	1.226	0.29	1.07 × 10^−2^
A0A078GPI5	Glycosyltransferase	1	Cytosol	Unknown	1.222	0.29	2.74 × 10^−2^
A0A078I909	Xyloglucan endotransglucosylase/hydrolase	5	Apoplast	Cell wall organization	1.22	0.29	2.97 × 10^−2^
A0A078J5T4	(rape) hypothetical protein	13	-	Unknown	1.218	0.28	3.66 × 10^−3^
A0A078JW32	(rape) hypothetical protein	11	-	Unknown	1.217	0.28	7.49 × 10^−3^
A0A078JR63	(rape) hypothetical protein	1	Cytosol	Protein folding	1.212	0.28	1.77 × 10^−2^
A0A078I708	GrpE protein homolog	5	Mitochondria	Protein transport	1.211	0.28	2.00 × 10^−2^
A0A078ISW8	(rape) hypothetical protein	4	-	Lignin biosynthesis	1.215	0.28	4.50 × 10^−2^
A0A078EQE4	Non-specific lipid-transfer protein	3	-	Lipid transport	1.21	0.27	4.62 × 10^−3^
A0A078H8M2	1,3-beta-glucan synthase	13	Cell membrane	Cell wall organization	1.205	0.27	2.14 × 10^−2^
A0A078GSR8	BnaA01g06570D protein	3	Vacuole	Vacuolar protein processing	1.209	0.27	2.51 × 10^−2^
A0A078F8D8	ABC-type xenobiotic transporter	6	-	Xenobiotics transport	1.204	0.27	2.84 × 10^−2^
A0A078IDI4	BnaA02g29390D protein	7	-	Unknown	1.208	0.27	3.40 × 10^−2^
A0A078JCY9	BnaC06g42330D protein	4	Nucleus	Proteolysis	1.207	0.27	4.61 × 10^−2^
A0A078HR88	(rape) hypothetical protein	6	-	Lipid metabolism	1.199	0.26	1.41 × 10^−2^
A0A078HYE9	(rape) hypothetical protein	10	Vacuole	Unknown	1.194	0.26	2.10 × 10^−2^
A0A078I7F1	Peroxidase	6	-	Redox homeostasis	1.194	0.26	2.10 × 10^−2^
A0A078I415	BnaC02g00800D protein	5	-	Protein folding	1.194	0.26	2.43 × 10^−2^
A0A078JE52	(rape) hypothetical protein	7	Chloroplast	Unknown	1.199	0.26	2.67 × 10^−2^
A0A078G6 × 1	BnaA02g08820D protein	1	Cytosol	Protein folding	1.197	0.26	3.84 × 10^−2^
A0A078GSE8	(rape) hypothetical protein	1	-	Redox homeostasis	1.192	0.25	1.82 × 10^−2^
P93063	(rape) hypothetical protein	6	-	Unknown	1.187	0.25	2.14 × 10^−2^
A0A078HWD9	(rape) hypothetical protein	9	-	Unknown	1.187	0.25	2.19 × 10^−2^
A0A078JHI7	(rape) hypothetical protein	1	Chloroplast	Photosynthetic light reactions	1.188	0.25	4.43 × 10^−2^
A0A078J7I5	Lipoxygenase	4	-	Lipid metabolism	1.181	0.24	2.21 × 10^−2^
A0A078GP42	BnaC09g36360D protein	1	Cytosol	Unknown	1.183	0.24	2.35 × 10^−2^
A0A078FL44	Chlorophyll a-b binding protein	3	Chloroplast	Photosynthetic light reactions	1.179	0.24	2.82 × 10^−2^
A0A078FPX0	BnaC05g00300D protein	3	Chloroplast	Ion transport	1.18	0.24	3.51 × 10^−2^
A0A078GHK3	1,3-beta-glucan synthase	4	Cell membrane	Cell wall organization	1.181	0.24	3.72 × 10^−2^
A0A078FGY6	BnaA03g01740D protein	6	-	Proteolysis	1.183	0.24	3.95 × 10^−2^
A0A078FTS5	Lipoxygenase	9	-	Lipid metabolism	1.176	0.23	3.26 × 10^−2^
A0A078JJT8	BnaC02g46730D protein	9	-	Unknown	1.173	0.23	3.58 × 10^−2^
A0A078FLH0	Inositol-3-phosphate synthase	2	Cytosol	Lipid metabolism	1.174	0.23	4.19 × 10^−2^
A0A078FAW1	Phospholipase A1	2	-	Lipid metabolism	1.173	0.23	4.21 × 10^−2^
A0A078IWD9	Branched-chain-amino-acid aminotransferase	3	Chloroplast	Amino acid metabolism	1.175	0.23	4.62 × 10^−2^
A0A078HXB3	BnaA05g32660D protein	4	-	Unknown	0.83	−0.27	4.79 × 10^−2^
A0A078FTG4	(rape) hypothetical protein	13	-	Unknown	0.821	−0.28	3.38 × 10^−2^
A0A078I495	(rape) hypothetical protein	16	-	Unknown	0.816	−0.29	2.16 × 10^−2^
A0A078GVN3	Carboxypeptidase	9	Apoplast	Proteolysis	0.817	−0.29	4.63 × 10^−2^
A0A078JCF7	BnaCnng39740D protein	8	Cell membrane	Unknown	0.81	−0.3	1.52 × 10^−2^
A0A078I1Z2	BnaA02g13970D protein	7	-	Carbohydrate metabolism	0.812	−0.3	1.70 × 10^−2^
A0A078GT00	BnaA05g31420D protein	15	-	Unknown	0.815	−0.3	2.07 × 10^−2^
A0A078JKB1	Pectin acetylesterase (Fragment)	9	-	Cell wall organization	0.809	−0.31	2.57 × 10^−2^
A0A078HZ31	Glyceraldehyde-3-phosphate dehydrogenase	4	-	Calvin Benson cycle	0.806	−0.31	2.68 × 10^−2^
A0A078H104	BnaA08g26240D protein	3	-	Unknown	0.807	−0.31	3.68 × 10^−2^
A0A078HA31	Carboxypeptidase	1	Apoplast	Proteolysis	0.8	−0.32	7.63 × 10^−3^
A0A078JBU5	(rape) hypothetical protein	6	Chloroplast	Chlorophyll metabolism	0.803	−0.32	8.23 × 10^−3^
A0A078F5V9	(rape) hypothetical protein	3	-	Unknown	0.802	−0.32	2.10 × 10^−2^
A0A078FN47	BnaA10g07360D protein	3	-	Unknown	0.803	−0.32	2.96 × 10^−2^
A0A078J6M0	BnaAnng16600D protein	10	-	Unknown	0.793	−0.33	4.30 × 10^−3^
A0A078I9C2	(rape) hypothetical protein	15	-	Amino acid metabolism	0.794	−0.33	4.54 × 10^−3^
A0A078GA58	(rape) hypothetical protein	6	Apoplast	Unknown	0.797	−0.33	5.64 × 10^−3^
A0A078I1U9	NAD(P)H dehydrogenase (quinone)	2	-	Redox homeostasis	0.794	−0.33	7.90 × 10^−3^
A0A078G1G3	BnaC02g24210D protein	2	Cytosol	Unknown	0.796	−0.33	8.66 × 10^−3^
A0A078FAN3	(rape) hypothetical protein	5	Nucleus	Coenzyme A biosynthesis	0.793	−0.33	1.37 × 10^−2^
A0A078J542	BnaA05g35220D protein	2	-	Unknown	0.795	−0.33	1.58 × 10^−2^
A0A078GHM5	BnaA03g59800D protein	5	-	Sulfate assimilation	0.795	−0.33	1.80 × 10^−2^
A0A078HA55	Succinate--CoA ligase [ADP-forming] subunit beta	1	Mitochondria	TCA cycle	0.797	−0.33	1.80 × 10^−2^
A0A078INE2	BnaA01g30370D protein	5	-	Proteolysis	0.794	−0.33	2.18 × 10^−2^
A0A078IYC4	BnaC04g53560D protein	4	Cytosol	Unknown	0.798	−0.33	2.46 × 10^−2^
A0A078HV59	BnaA03g53100D protein	13	-	Proteolysis	0.789	−0.34	3.43 × 10^−3^
A0A078I103	Starch synthase	5	Chloroplast	Starch biosynthesis	0.788	−0.34	1.93 × 10^−2^
A0A078IMS4	(rape) hypothetical protein	4	-	Unknown	0.792	−0.34	3.21 × 10^−2^
A0A078GXJ1	Glutathione hydrolase	7	Cell membrane	Redox homeostasis	0.785	−0.35	4.10 × 10^−3^
A0A078F6Z3	BnaA02g05770D protein	6	Cytosol	Unknown	0.783	−0.35	7.57 × 10^−3^
A0A078FRX3	(rape) hypothetical protein	2	Chloroplast	Photosynthetic light reactions	0.785	−0.35	8.26 × 10^−3^
Q42625	Glutamine synthetase	7	Cytosol	Amino acid metabolism	0.78	−0.36	2.72 × 10^−3^
A0A078J1J0	(rape) hypothetical protein	3	Plasmodesmata	Defense response	0.778	−0.36	4.22 × 10^−2^
A0A078GXM3	(rape) hypothetical protein	1	Chloroplast	Unknown	0.777	−0.36	4.50 × 10^−2^
A0A078HMR7	Ferredoxin--NADP reductase	10	Chloroplast	Photosynthetic light reactions	0.776	−0.37	9.90 × 10^−4^
A0A078HXA1	NAD(P)H dehydrogenase (quinone)	4	-	Redox homeostasis	0.776	−0.37	1.75 × 10^−3^
A0A078GBH5	(rape) hypothetical protein	2	Cell membrane	Unknown	0.773	−0.37	3.32 × 10^−2^
A0A078IJ48	Apyrase	3	-	Nucleoside diphosphate metabolism	0.772	−0.37	3.34 × 10^−2^
A0A078JFA9	(rape) hypothetical protein	2	Apoplast	Unknown	0.769	−0.38	2.51 × 10^−3^
A0A078FBH3	Histone H2A	1	Nucleus	Chromatin organization	0.768	−0.38	1.31 × 10^−2^
A0A078IUE0	Histone H2A	1	Nucleus	Chromatin organization	0.771	−0.38	3.58 × 10^−2^
A0A078GPW5	(rape) hypothetical protein	2	-	Unknown	0.763	−0.39	2.21 × 10^−2^
A0A078JIB2	BnaC08g46420D protein	1	-	Unknown	0.762	−0.39	2.32 × 10^−2^
A0A078J451	(rape) hypothetical protein	3	Cytosol	Unknown	0.764	−0.39	2.84 × 10^−2^
A0A078F9R8	Ferritin	6	Chloroplast	Ion transport	0.755	−0.4	3.02 × 10^−4^
A0A078GVU9	(rape) hypothetical protein	5	-	Unknown	0.76	−0.4	5.84 × 10^−4^
A0A078GH45	Alanine transaminase	1	-	Photorespiration	0.76	−0.4	2.78 × 10^−2^
A0A078F8F7	(rape) hypothetical protein	6	Nucleus	Chromosome condensation	0.75	−0.41	8.26 × 10^−5^
A0A078F4V7	Pectinesterase	3	-	Cell wall organization	0.754	−0.41	2.10 × 10^−2^
A0A078HKL0	BnaA07g02120D protein	2	Cytosol	Translation	0.746	−0.42	7.23 × 10^−4^
A0A078FQN7	(rape) hypothetical protein	1	-	Unknown	0.747	−0.42	3.01 × 10^−2^
A0A078JI07	(rape) hypothetical protein	1	Chloroplast	Rubisco assembly	0.746	−0.42	4.76 × 10^−2^
A0A078IZV0	(rape) hypothetical protein	8	Apoplast	Unknown	0.744	−0.43	9.07 × 10^−5^
A0A078J6D0	BnaC04g53030D protein	2	-	Cell wall organization	0.737	−0.44	5.14 × 10^−3^
A0A078FS35	(rape) hypothetical protein	1	Cytosol	Translation	0.738	−0.44	6.81 × 10^−3^
A0A078IRE8	(rape) hypothetical protein	2	-	Unknown	0.726	−0.46	4.78 × 10^−6^
A0A078G019	(rape) hypothetical protein	1	Chloroplast	Photosynthetic light reactions	0.726	−0.46	1.27 × 10^−5^
A0A078HZN5	Expansin	2	-	Cell wall organization	0.724	−0.47	5.78 × 10^−4^
A0A078I7K4	BnaC02g22590D protein	2	Vacuole	Unknown	0.724	−0.47	1.87 × 10^−3^
A0A078I0P0	BnaC05g36680D protein	2	Plasmodesmata	Signaling	0.717	−0.48	1.75 × 10^−3^
A0A078HMK9	(rape) hypothetical protein	1	-	Unknown	0.711	−0.49	1.44 × 10^−2^
A0A078FB79	BnaA02g05290D protein	8	Nucleus	Defense response	0.706	−0.5	6.87 × 10^−7^
A0A078GTF2	Non-specific serine/threonine protein kinase	2	-	Signaling	0.7	−0.52	6.41 × 10^−4^
A0A078HAP6	BnaA02g30740D protein	3	-	Cell wall organization	0.691	−0.53	9.09 × 10^−7^
A0A078GNZ7	Glycine cleavage system H protein	2	Mitochondria	Photorespiration	0.694	−0.53	1.35 × 10^−6^
A0A078F776	Histone H2B	1	Nucleus	Chromatin organization	0.694	−0.53	1.93 × 10^−2^
A0A078JLL8	BnaC01g44910D protein	1	-	Lipid transport	0.691	−0.53	2.63 × 10^−2^
A0A078I390	(rape) hypothetical protein	1	-	Unknown	0.69	−0.54	8.04 × 10^−3^
A0A078HLM5	(rape) hypothetical protein	2	-	Unknown	0.666	−0.59	6.50 × 10^−5^
A0A078F5L4	(rape) hypothetical protein	2	Plasmodesmata	Signaling	0.659	−0.6	9.65 × 10^−6^
A0A078JG76	Biotin carboxyl carrier protein of acetyl-CoA carboxylase	1	Chloroplast	Lipid metabolism	0.661	−0.6	1.45 × 10^−2^
A0A078G4U7	(rape) hypothetical protein	1	-	Unknown	0.652	−0.62	4.49 × 10^−3^
A0A078I2L1	BnaC02g41300D protein	2	Plasmodesmata	defense response	0.631	−0.66	2.53 × 10^−6^
A0A078I8 × 8	(rape) hypothetical protein	1	Chloroplast	Translation	0.625	−0.68	8.50 × 10^−9^
A0A078G384	DNA polymerase	1	Nucleus	DNA repair and replication	0.61	−0.71	1.15 × 10^−7^
A0A078HN54	(rape) hypothetical protein	1	-	Unknown	0.611	−0.71	1.42 × 10^−4^
A0A078GRB9	Pectinesterase	2	-	Cell wall organization	0.568	−0.82	1.19 × 10^−9^
A0A078HF73	BnaC03g41590D protein	2	-	Unknown	0.561	−0.83	1.36 × 10^−10^
A0A078F748	BnaC08g11470D protein	1	-	Unknown	0.56	−0.84	1.29 × 10^−5^
A0A078GMM9	(rape) hypothetical protein	1	-	Unknown	0.364	−1.46	2.64 × 10^−15^
A0A078HA23	(rape) hypothetical protein	1	-	Protein transport	0.275	−1.86	2.64 × 10^−15^

**Table A2:** Relative abundance of identified bacterial phyla in soil of rapeseed plants with amendment application (AM) and non-treated plants (Control). Values are means ± S.E (*n* = 3), values with different letter and cell colour indicate that values are significantly different (*p* < 0.05) *t*-test.

	Control	AM
Richness (nº OTUs)	283 ± 52 a	317 ± 65 a
Actinobacteriota	0.057 ± 0.016 b	0.121 ± 0.009 a
Bacteroidota	0.068 ± 0.002 a	0.077 ± 0.004 a
Bdellovibrionota	0.010 ± 0.004 a	0.005 ± 0.003 a
Chloroflexi	0.068 ± 0.014 a	0.083 ± 0.004 a
Cyanobacteria	0.042 ± 0.012 a	0.027 ± 0.014 a
Fibrobacterota	0.002 ± 0.001 a	0.001 ± 0.001 a
Firmicutes	0.008 ± 0.001 a	0.018 ± 0.005 a
Gemmatimonadota	0.024 ± 0.004 a	0.022 ± 0.004 a
Myxococcota	0.014 ± 0.005 a	0.017 ± 0.004 a
Nitrospirota	0.013 ± 0.001 a	0.010 ± 0.001 a
Patescibacteria	0.107 ± 0.031 a	0.033 ± 0.009 a
Planctomycetota	0.162 ± 0.017 a	0.198 ± 0.017 a
Proteobacteria	0.178 ± 0.024 a	0.164 ± 0.012 a
Verrucomicrobiota	0.087 ± 0.003 a	0.078 ± 0.004 a
